# Surrogate gradients for analog neuromorphic computing

**DOI:** 10.1073/pnas.2109194119

**Published:** 2022-01-14

**Authors:** Benjamin Cramer, Sebastian Billaudelle, Simeon Kanya, Aron Leibfried, Andreas Grübl, Vitali Karasenko, Christian Pehle, Korbinian Schreiber, Yannik Stradmann, Johannes Weis, Johannes Schemmel, Friedemann Zenke

**Affiliations:** ^a^Kirchhoff-Institute for Physics, Heidelberg University, 69120 Heidelberg, Germany;; ^b^Computational Neuroscience Group, Friedrich Miescher Institute for Biomedical Research, 4058 Basel, Switzerland

**Keywords:** neuromorphic hardware, recurrent neural networks, spiking neural networks, surrogate gradients, self-calibration

## Abstract

To rapidly process temporal information at a low metabolic cost, biological neurons integrate inputs as an analog sum, but communicate with spikes, binary events in time. Analog neuromorphic hardware uses the same principles to emulate spiking neural networks with exceptional energy efficiency. However, instantiating high-performing spiking networks on such hardware remains a significant challenge due to device mismatch and the lack of efficient training algorithms. Surrogate gradient learning has emerged as a promising training strategy for spiking networks, but its applicability for analog neuromorphic systems has not been demonstrated. Here, we demonstrate surrogate gradient learning on the BrainScaleS-2 analog neuromorphic system using an in-the-loop approach. We show that learning self-corrects for device mismatch, resulting in competitive spiking network performance on both vision and speech benchmarks. Our networks display sparse spiking activity with, on average, less than one spike per hidden neuron and input, perform inference at rates of up to 85,000 frames per second, and consume less than 200 mW. In summary, our work sets several benchmarks for low-energy spiking network processing on analog neuromorphic hardware and paves the way for future on-chip learning algorithms.

## Significance

Neuromorphic systems aim to accomplish efficient computation in electronics by mirroring neurobiological principles. Taking advantage of neuromorphic technologies requires effective learning algorithms capable of instantiating high-performing neural networks, while also dealing with inevitable manufacturing variations of individual components, such as memristors or analog neurons. We present a learning framework resulting in bioinspired spiking neural networks with high performance, low inference latency, and sparse spike-coding schemes, which also self-corrects for device mismatch. We validate our approach on the BrainScaleS-2 analog spiking neuromorphic system, demonstrating state-of-the-art accuracy, low latency, and energy efficiency. Our work sketches a path for building powerful neuromorphic processors that take advantage of emerging analog technologies.

In recent years, deep artificial neural networks (ANNs) have surpassed human-level performance on many difficult tasks (1–3). The human brain, however, remains unchallenged in terms of its energy efficiency and fault tolerance. A fundamental property underlying these capabilities is spatiotemporal sparseness (4), which is directly linked to the way biological spiking neural networks (SNNs) process and exchange information. Spiking neurons receive and integrate inputs on their analog membrane potentials and, upon reaching the firing threshold, emit action potentials, or spikes. These binary events propagate asynchronously through the SNN and are ultimately received by other neurons.

Neuromorphic engineering attempts to mirror the power efficiency and robustness of the brain by replicating its key architectural properties (5–9). Here, one distinguishes between fully digital, analog, and mixed-signal systems. Digital systems “simulate” the analog dynamics of spiking neurons, e.g., their membrane potentials (10–15). In contrast, analog and mixed-signal solutions “emulate” neuronal or synaptic dynamics and states by representing them as physical voltages, currents, or conductance changes evolving in continuous time (7, 13, 14, 16). Thus, by explicitly taking advantage of physical properties and dynamics of the underlying hardware substrate, neuromorphic computing holds the key to building power-efficient and scalable SNNs in silico (15, 19, 20).

However, to serve meaningful computational purpose, these analog devices require training. The most successful training schemes for ANNs are gradient-based. Yet, extending similar training techniques to SNNs and neuromorphic hardware poses several challenges. First, one has to overcome the binary nature of spikes, which impedes vanilla gradient descent (21–23). Second, training has to ensure sparse spiking activity to exploit the superior power efficiency of SNN processing (24, 25). Finally, training has to achieve all of the above while coping with analog hardware imperfections inevitably tied to their manufacturing process.

In this article, we tackle the above challenges by extending previous work on surrogate gradients, which have emerged as a powerful method for training SNNs end-to-end (23). Specifically, we developed an in-the-loop (ITL) training framework for surrogate gradient learning and applied it to the mixed-signal BrainScaleS-2 single-chip system (26–28). We demonstrate that SNNs trained using our approach solve several challenging benchmark problems by taking advantage of sparse, precisely timed spikes instead of firing rates. The resulting SNNs reach comparable accuracy levels to corresponding software simulations and perform energy-efficient inference with ultralow latency by taking full advantage of BrainScaleS’ accelerated nature and in-memory compute capabilities. Crucially, we show that ITL surrogate gradients achieve this through self-calibration, whereby training automatically corrects for device mismatch without the need for costly offline calibration.

## The BrainScaleS-2 Analog Neuromorphic Substrate

In this article, we relied on the analog BrainScaleS-2 single-chip system. It features 512 analog neuron circuits, whose dynamics obey the leaky integrate-and-fire (LIF) equation

[1] $$C\frac{\text{d}V}{\text{d}t}=-g_{\text{leak}}\left(V-V_{\text{leak}}\right)+I,$$

which can optionally be augmented by adaptation currents and an exponential spiking nonlinearity. The membrane potential *V* is explicitly represented on the chip as an analog voltage measured across a capacitor and evolves continuously in time. The leak conductance $g_{\text{leak}}$ pulls the membrane toward the leak potential $V_{\text{leak}}$, resulting in an exponential decay with time constant $\tau_{\text{m}}\equiv C/g_{\text{leak}}$. Due to the substrate’s small intrinsic capacitances and comparatively large currents, the dynamics of the spiking neurons implemented on BrainScaleS-2 evolve 10^3^ times faster than biological neurons.

**Fig. 1.**
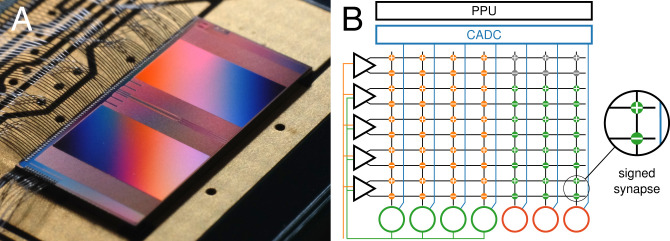
The mixed-signal BrainScaleS-2 chip. (*A*) Close-up chip photograph. (*B*) Implementation of a multilayer network on the analog neuromorphic core. Input spike trains are injected via synapse drivers (triangles) and relayed to the hidden-layer neurons (green circles) via the synapse array. Spikes in the hidden layer are routed on-chip to the output units (red circles). Each connection is represented by a pair of excitatory and inhibitory hardware synapses, which holds a signed weight value. The analog membrane potentials are read out via the CADC and further processed by the PPU.

Whenever the membrane potential crosses the firing threshold ϑ, an outgoing spike is generated, and the membrane is reset. An on-chip event router propagates both internally generated and external spikes to connected neurons, allowing it to form feed-forward as well as recurrent topologies. To that end, each neuron integrates stimuli from a column of 256 synapses, each with a 6-bit weight stored in local static random-access memory. The resulting postsynaptic currents *I*, which are integrated on the membrane capacitor, follow an exponential time course similar to the membrane dynamics themselves. The sign of a synapse is determined as a presynaptic property. However, we allowed for a continuous transition between positive and negative weights during training by merging synapse circuits of opposing signs (Fig. 1*B*).

BrainScaleS-2 allows individually adjusting all neuronal parameters, including reference potentials and time constants, on a per-neuron basis to flexibly emulate different target dynamics. This fine-grained control also facilitates calibration to mitigate deviations induced by variations in the production process. In this article, we, however, make use of this parameterization to actively decalibrate the system, thereby allowing us to systematically explore self-calibration properties of our learning algorithm.

## ITL Surrogate Gradients on Analog Hardware

To train SNNs on BrainScaleS-2, we developed a general learning framework to optimize recurrent and multilayer networks. Our approach is based on the notion of surrogate gradients, which overcome vanishing gradients and critical points associated with nondifferentiable spiking dynamics (23). Surrogate gradient learning flexibly supports arbitrary differentiable loss functions and can seamlessly exploit both rate-based and spike timing-based coding schemes.

**Fig. 2.**
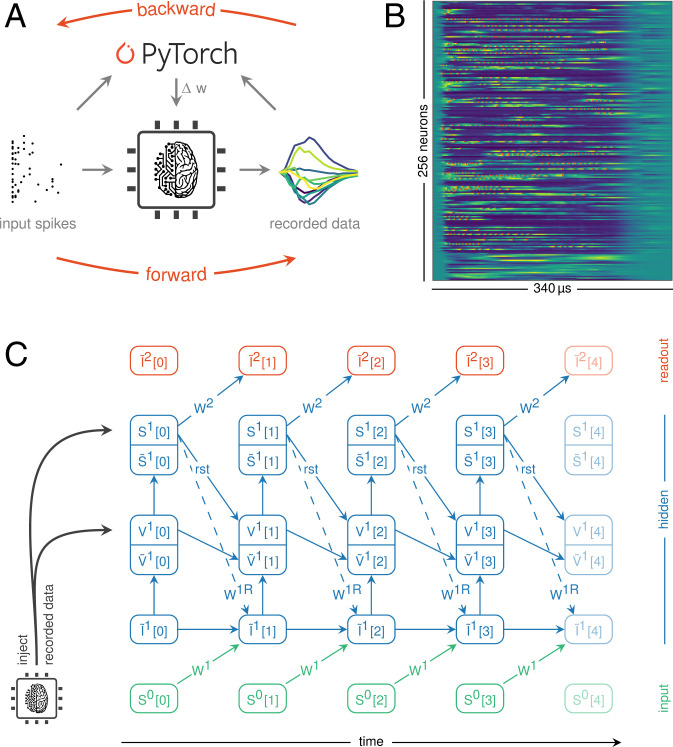
Surrogate gradient learning on BrainScaleS-2. (*A*) Illustration of our ITL training scheme. The forward pass is emulated on the BrainScaleS-2 chip. Observables from the neuromorphic substrate as well as the input spike trains are processed on a conventional computer to perform the backward pass. The calculated weight updates are then written to the neuromorphic system. (*B*) Parallel recording of analog traces and spikes from 256 neurons via the CADC. (*C*) The differentiable computation graph results from the integration of LIF dynamics. The time dimension is unrolled from left to right, and information flows from bottom to top within an integration step. Synaptic currents are derived from the previous layer’s spikes and potential recurrent connections, multiplied by the respective weights (*W*). Stimuli are integrated on the neurons’ membranes (*V*), which trigger spikes (*S*) upon crossing their thresholds. These observables are continuously synchronized with data recorded from the hardware. Spikes as well as reset signals (rst) are propagated to the next time step, which also factors in the decay of currents and potentials.

Broadly, our ITL approach works as follows (Fig. 2): First, we emulate the forward pass on the analog neuromorphic substrate and record both spikes and internal membrane traces (Fig. 2 *A* and *B*). By injecting the latter into an otherwise approximate software model, we effectively render the neuromorphic SNN differentiable. This permits the evaluation of surrogate gradients and the calculation of weight updates using backpropagation through time (BPTT) on graphics-processing unit–enabled autodifferentiation libraries (29), in combination with state-of-the-art optimizers. At the same time, our learning algorithm self-corrects for parameter mismatch of the analog components (Fig. 2*C*). Finally, we close the loop by transferring the updated weights back to the analog system.

In the following, we elaborate on the two central steps, namely, the recording of data from the neuromorphic system and their integration into the computation graph.

### Recording Spikes and Analog Membrane Traces

Surrogate gradient learning crucially relies on the neurons’ membrane potentials. On an analog system like BrainScaleS-2, these are represented as physical voltages and are hence not readily available for numerical computation. The required digitization is often challenging due to the inherent parallelism of these substrates. This bottleneck is further emphasized by accelerated systems.

BrainScaleS-2 solves this problem by incorporating column-parallel analog-to-digital converters (CADCs) to simultaneously digitize the membrane potentials of all neurons (Fig. 1*B*). We trigger the ADC conversions via the embedded plasticity processing units (PPUs) (30) to ensure higher and more stable sampling rates compared to a host-based scheduling. This furthermore enables the implementation of a fast inference mode, where only classification results are transmitted to the host. When training the network, however, each recorded sample is instantly transferred to an intermediate external memory region, from where it is asynchronously read by the host machine at the end of an input pattern or batch. In total, we reach a sample rate of ∼0.6 MSample×s^–1^, corresponding to a sampling interval of 1.7 µs. For 256 neurons, this yields a total data rate of 1.2 Gbit×s^–1^. In addition to the sampled membrane traces, we continuously record and time-stamp the spike events emitted by the substrate.

### A Computation Graph for Analog Circuits

To compute weight updates based on surrogate gradients, we incorporate these aggregated data into a computation graph that approximates the underlying neuronal dynamics on the neuromorphic substrate. To that end, we iteratively simulate the neuronal dynamics to obtain the graph in which we inject the actual recorded membrane traces. Thus, we use measured quantities, where available, and only rely on the model estimates for internal variables that are not measured, e.g., the synaptic currents.

We formulate the graph on a regular time grid of time step Δt derived from the sampling period of the membrane traces. Although the spike trains from the neuromorphic substrate are known with much higher temporal resolution, they are also aligned to these bins. Depending on the coding scheme and network topology, an increased resolution can be beneficial and allow us to better capture causal relations between spikes. In this case, the computation graph can be evaluated on a finer time scale and, for that purpose, operate on interpolated membrane traces.

To reconstruct the internal states, we start by assuming ideal LIF dynamics (Eq. **1**), which we numerically integrate by taking into account its temporal decay and the calculated synaptic currents $\tilde{I}[t]$, which, in turn, are based on the presynaptic spikes ${\tilde{S}}_{j}[t]$ of neuron *j*:

[2] $$\tilde{V}[t+1]=\tilde{V}[t]\cdot {\text{e}}^{-\Delta t/\tau_{\text{m}}}+\tilde{I}[t],$$

[3] $$\tilde{I}[t+1]=\tilde{I}[t]\cdot {\text{e}}^{-\Delta t/\tau_{\text{s}}}+\sum_{j}W_{j}{\tilde{S}}_{j}[t].$$

For brevity, we consider a dimensionless formulation of the LIF dynamics, in which we assume a leak potential $V_{\text{leak}}=0$, a capacitance *C* = 1, and a firing threshold ϑ=1 (23). Physical variables can be readily obtained through appropriate rescaling (cf. *Materials and Methods* and *SI Appendix*). Eq. **3** can be augmented by an additional term to encompass recurrent connections. The modeled state variables, indicated by the tilde ($\tilde{}$), represent the estimates of the on-chip dynamics. Since these can deviate from the actual emulation and hence distort the resulting gradients, we, in their place, insert the normalized recorded data. For this purpose, we introduce an auxiliary identity function $f(x,\tilde{x})\equiv x$ and define surrogate derivatives ∂f/∂x=0 and $\partial f/\partial \tilde{x}=1$. Eq. **2** can now be modified to

[4] $$\tilde{V}[t+1]=f\left(V[t+1],\tilde{V}[t]\cdot {\text{e}}^{-\Delta t/\tau_{\text{m}}}+\tilde{I}[t]\right)\;.$$

A similar approach is taken for spikes by defining ${\tilde{S}}_{j}[t](S_{j}[t],{\tilde{V}}_{j}[t])\equiv S_{j}[t]$ with associated derivatives

[5] $$\frac{\partial {\tilde{S}}_{j}[t]}{\partial S_{j}[t]}=0,\frac{\partial {\tilde{S}}_{j}[t]}{\partial {\tilde{V}}_{j}[t]}={\left(\beta \cdot |{\tilde{V}}_{j}[t]-\vartheta |\right)}^{-2},$$

where *β* describes the steepness of the surrogate gradient (31).

When performing the backward pass and, to this end, calculating $\partial L/\partial \theta =\ldots \partial \tilde{S}/\partial \tilde{V}\cdot \partial \tilde{V}/\partial \theta$, the sampled values for the membrane potential are used whenever an expression containing $\tilde{V}$ is evaluated, e.g., in $\partial \tilde{S}/\partial \tilde{V}$. The estimates, in contrast, are used to determine further derivatives $\partial \tilde{V}/\partial \theta$, which occur in the recursion relation of BPTT.

### Flexible Choice of Loss Functions

The suggested framework allows us to operate on any differentiable loss that can be formulated on the data acquired from the neuromorphic system. This encompasses loss functions based on the spiking activity of the neurons, as well as on their membrane voltages (cf. *Materials and Methods*). The task-specific loss can be augmented by regularization functions. These might, on one hand, target an improved generalization performance or, on the other hand, an adaptation to hardware-specific constraints, such as finite weights and dynamic ranges of analog signals. Such terms can furthermore be directly tailored to shape the activity of the emulated SNNs and result in sparse firing patterns.

## Results

We trained BrainScaleS-2 on a series of spike-based vision and speech-recognition tasks using our ITL learning framework. Specifically, we chose classification tasks requiring evidence integration on widely different time scales, which allowed us to probe the efficiency of our approach on both feed-forward and recurrent network topologies.

First, we trained a feed-forward network consisting of a single hidden layer with 246 neurons on the Modified National Institute of Standards and Technology (MNIST) dataset (32). To accommodate the data to a fan-in of 256 inputs, we reduced the original 28 × 28 images to 16 × 16 pixels. We then converted the pixels into a spike-latency code (cf. *Materials and Methods*). The network was optimized by using the Adam optimizer (33) to minimize a max-over-time loss $L=\text{NLL}(\text{softmax}(\max_{t}\;V_{i}^{\text{O}}[t]),y^{⋆})$, with the negative log-likelihood (NLL), the membrane traces of the output layer $V_{i}^{\text{O}}[t]$, and the true labels $y^{⋆}$. To prevent excessive amplitudes and, in turn, clipping of $V_{i}^{\text{O}}$ on the analog substrate, we included a penalty $\rho_{\text{a}}\cdot {\text{mean}}_{i}({\left(\max_{t}V_{i}^{\text{O}}[t]\right)}^{2})$. We furthermore added an activity-shaping term to promote sparse activity patterns (cf. Eq. **6**). Notably, this contribution could only reduce the network’s activity and did not act as an upward-pulling homeostatic force. Being based on surrogate gradients, our approach nevertheless allowed training the network starting from a quiescent hidden layer.

**Fig. 3.**
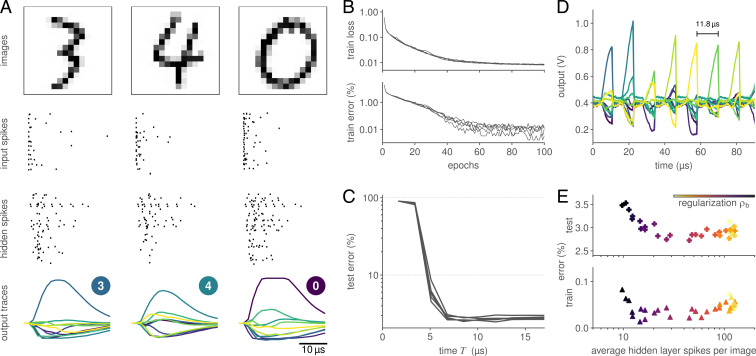
Classification of the MNIST dataset. (*A*) Three snapshots of the SNN activity, consisting of the downscaled 16 × 16 input images (*Top*), spike raster of both the input spike trains and hidden-layer activity (*Middle*), and readout neuron traces (*Bottom*). The latter show a clear separation, and, hence, a correct classification of the presented images. (*B*) Loss and accuracy over the course of 100 training epochs for five initial conditions. (*C*) The time to decision is consistently below 10 µs. Here, the classification latency was determined by iteratively reevaluating the max-over-time for output traces (see *A*) restricted to a limited interval [0,T]. (*D*) This low latency allowed us to inject an image every 11.8 µs, corresponding to more than 85,000 classifications per second. This was achieved by artificially resetting the state of the neuromorphic network in between samples. (*E*) The neuromorphic system can be trained to perform classification with sparse activity. When sweeping the regularization strength, a state of high performance was evidenced over more than an order of magnitude of hidden-layer spike counts.

During training, the neuromorphic substrate learned to correctly infer and represent the correct class memberships as the maximally responsive output units (Fig. 3 *A* and *B*). Interestingly, the inhibition of the other units was not explicitly demanded by the loss function, but emerged naturally through optimization. After 100 epochs, the model almost perfectly fit the training samples and achieved an overall accuracy of 97.2 ± 0.1% on held-out test data (Table 1). We were able to reduce overfitting by augmenting the data through random rotations of up to 15^∘^. Dropout similarly improved test performance, and combining it with data augmentation resulted in an accuracy of 97.6 ± 0.1% on BrainScaleS-2.

**Table 1.** Comparison of results achieved with networks trained on BrainScaleS-2 and in software as well as an ANN baseline

	Implementation	Remarks	Accuracy, %	
			Train	Test
16× 16 MNIST	BSS-2		100.0 ± 0.0	97.2 ± 0.1
	BSS-2	Dropout + rotation	97.3 ± 0.1	97.6 ± 0.1
	Software		100.0 ± 0.0	97.5 ± 0.1
	Software	Dropout + rotation	97.7 ± 0.1	98.0 ± 0.0
	Reference ANN		100.0 ± 0.0	98.1 ± 0.1
	Reference ANN	Dropout + rotation	99.0 ± 0.0	98.7 ± 0.1
16× 16 F-MNIST	BSS-2		90.1 ± 0.6	84.2 ± 0.2
	Software		95.2 ± 0.1	85.5 ± 0.1
	Reference ANN		97.9 ± 0.1	88.0 ± 0.2
SHD	BSS-2		96.6 ± 0.5	76.2 ± 1.3
	BSS-2	Augmentation	90.7 ± 0.5	80.6 ± 1.0
	Software		100.0 ± 0.0	71.2 ± 0.3
	Software	Augmentation	90.9 ± 0.2	79.9 ± 0.7

As a comparison, we trained the same SNN purely in software and, in that process, ignored all hardware-specific constraints, including the finite weight resolution. With an accuracy of 97.5 ± 0.1% on the test data, the software implementation only slightly surpassed BrainScaleS-2. As a baseline for the downscaled 16 × 16 MNIST dataset, we furthermore trained an equivalently sized ANN with rectified linear units, which resulted in an accuracy of 98.1 ± 0.1%. Dropout as well as augmentation again improved upon these numbers, resulting in a best-effort performance of 98.7 ± 0.1%. Importantly, these accuracy figures—within their uncertainties—resembled results on the full-size MNIST images, suggesting that these two datasets are comparable in their complexity.

To further explore the computational abilities of BrainScaleS-2 trained with surrogate gradients, we used the same network architecture to classify 16 × 16 Fashion-MNIST (34), which resulted in a test accuracy of 84.2 ± 0.2% (Table 1).

### Low-Latency Neuromorphic Computation

The output traces of trained networks suggested that for latency-encoded inputs, as used above, the decision is available long before the end of a stimulus (cf. Fig. 3*A*). To determine the network’s classification latency, we artificially restricted the readout layer’s membrane traces (cf. Fig. 3*A*) to varying time intervals [0,T], over which we based the network’s decision, as given by the maximally active unit. We found that the readout reached its peak accuracy within 8 µs after the first input spike (Fig. 3*C*).

Low classification latency, however, does not automatically translate into high inference rates, but is also affected by the neuronal and synaptic time constants. These time constants determine the rate by which state variables decay back to baseline within the neuron circuits and, hence, impose a minimum separation of independent stimuli. Still, to translate low classification latency into high inference rates, we added an artificial reset of the neuromorphic units 10 µs after inserting the first input spike. Specifically, we exploited a feature of BrainScaleS-2 that allowed us to concurrently reset the analog membrane circuits and clamped all synaptic currents to their respective baselines (Fig. 3*D*). This allowed us to infer images with a separation of 11.8 µs, allowing our SNNs to accurately classify more than 85,000 (85 k) images per second with a latency of 8 µs.

Moreover, we measured the system’s power consumption. When emulating the trained SNN, the full BrainScaleS-2 chip consumed ∼200 mW. This figure included the current draw from the analog neuromorphic core, the plasticity processors, all surrounding periphery, and the high-speed communication links. Combining this measurement with the above throughput results in an energy consumption of 2.4 µJ per classified image.

### Efficiency through Sparse Spiking Activity

A key advantage of SNNs is their sparse temporal spiking activity, which is presumed crucial for the power efficiency of the brain (4). For similar reasons, it is also important for larger neuromorphic systems and particularly in scenarios in which several chips cooperate by exchanging spikes over communication channels with limited bandwidth.

To ensure sparse activity on the BrainScaleS-2 system, we augmented the training loss by a regularization term

[6] $$L_{\text{reg}}=\rho_{\text{b}}\frac{1}{N_{\text{H}}}\sum_{i=1}^{N_{\text{H}}}{\left(\sum_{t}S_{i}^{\text{H}}[t]\right)}^{2},$$

with the strength parameter $\rho_{\text{b}}$, the hidden-layer size $N_{\text{H}}$, and the corresponding hidden-layer spike trains $S_{i}^{\text{H}}$ (35). We trained the above feed-forward SNNs for a range of different values $\rho_{\text{b}}$ and measured both their accuracy and average hidden-layer spike counts. All resulting network configurations were able to fit the training data with high accuracy (Fig. 3*E*). More importantly, they reached a constant test accuracy of 97.2% for activity levels down to ∼20 hidden-layer spikes per image. When only using 10 spikes on average, we observed a slight decrease in performance. At such low spike counts, the networks operated in a regime far from the rate-coding limit and, hence, had to rely on individual spikes and their timing.

### Self-Calibration through ITL Learning

**Fig. 4.**
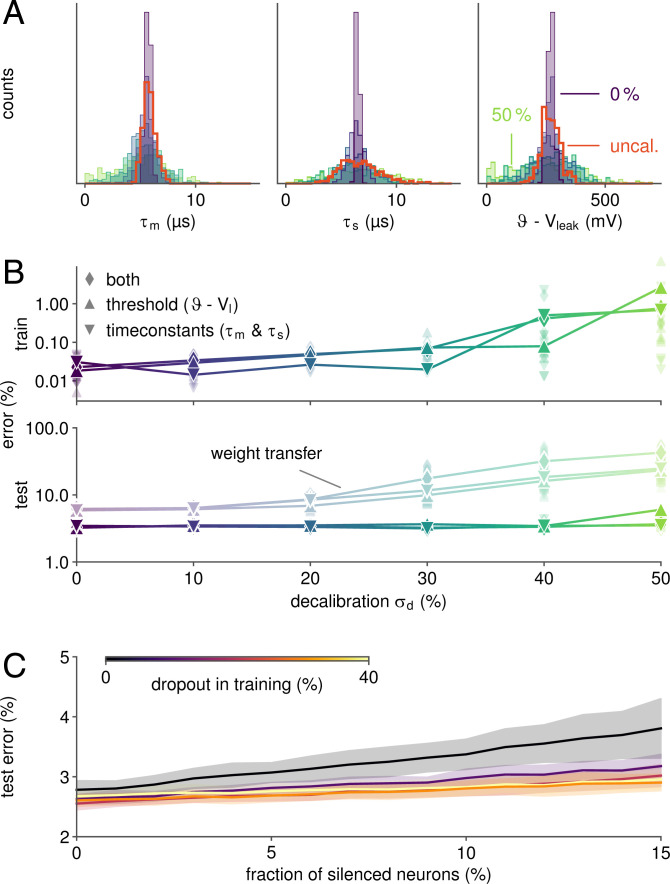
Self-calibration and robust performance on inhomogeneous substrates. (*A*) Distribution of measured neuronal parameters for various degrees of decalibration in the range of 0 to 50%. For this purpose, the analog circuits were deliberately detuned toward individual target values drawn from normal distributions of variable widths. Distributions for uncalibrated (uncal.) parameters are shown in red. (*B*) Despite assuming homogeneously behaving circuits in the computation graph, ITL training widely compensated the fixed-pattern deviations shown in *A*. In contrast, simply loading a software-trained network results in an increased test error, especially for a strong decalibration $\sigma_{\text{d}}$. For configurations with extreme mismatch, some networks suffered from dysfunctional states (e.g., leak-over-threshold). (*C*) When incorporating dropout regularization during training, networks become widely resilient to failure of hidden neurons.

The above results were obtained with a calibrated BrainScaleS-2 system in which the parameter deviations due to device mismatch were largely compensated, and the computation graph hence closely matched the emulated dynamics. Nevertheless, a certain degree of residual mismatch remained. To quantify whether and how well our ITL scheme self-calibrates the substrate during learning, we performed a series of additional experiments, in which we deliberately decalibrated the system. Specifically, we calibrated each neuron’s time constants and threshold to individual target values. These were drawn from normal distributions with a mean corresponding to the original calibration targets. We generated parameter sets by varying their normalized SDs $\sigma_{\text{d}}$ in the range of 0 to 50% (Fig. 4*A*). This notably exceeded the mismatch present on an uncalibrated BrainScaleS-2 system. To dissect the influence of poorly matching time constants and misaligned thresholds, we first detuned $\tau_{\text{m,s}}$ and $\vartheta -V_{\text{leak}}$ separately and, finally, all of these parameters at the same time. Each of these experiments was repeated for five random seeds.

For each set of parameters, we trained the SNN on the neuromorphic system, still assuming ideal dynamics when constructing the computation graph, as done previously. In other words, we explicitly ignored the introduced mismatch. Nevertheless, learning performance was hardly affected by decalibration up to $\sigma_{\text{d}}=30\%$. Beyond that point, training error levels remained low, but gradually increased (Fig. 4*B*). The testing performance, however, was (except for the highest $\sigma_{\text{d}}$) unaffected by the artificial mismatch. Notably, for high decalibration levels, some network configurations suffered from pathological network states, which were caused by some neurons entering a suprathreshold regime without external input. Thus, even for mismatch levels far above the ones expected for BrainScaleS-2 and similar systems, ITL learning effectively self-calibrated the analog neuromorphic SNNs. To illustrate the added benefit of such self-calibration, we also established the baseline performance for weight transfer, whereby networks were trained in software, and the weights were transferred to the neuromorphic chip subsequently. While a performance gap between ITL and weight transfer was already noticeable for the calibrated system, higher decalibration levels $\sigma_{\text{d}}$ dramatically widened this gap (Fig. 4*B*).

### Training for Robustness

We furthermore investigated the resilience of trained SNNs to defects occurring after deployment, e.g., failing neuron circuits. To this end, we simulated neuronal death by artificially silencing randomly selected units in the hidden layer of the network after training. As expected, performance deteriorated with an increasing fraction of disabled neurons (Fig. 4*C*).

However, when robustness was encouraged during training using dropout, the resilience to such neuronal failures was largely improved. For networks trained with a dropout rate of 40%, the test error increased by only 10% when silencing 15% of the hidden-layer units. In contrast, it grew by 37% when dropout was not used during training.

### Speech Recognition with Recurrent SNNs

So far, our analysis was limited to tasks with short time horizons, which can be readily solved using feed-forward networks. But other tasks, such as speech recognition or keyword spotting, may require working memory and thus recurrent architectures. On BrainScaleS-2, recurrent connectivity is readily supported by a flexible event router. Further, recurrence is easily integrated into our ITL learning scheme by adding recurrent connections to Eq. **3**.

**Fig. 5.**
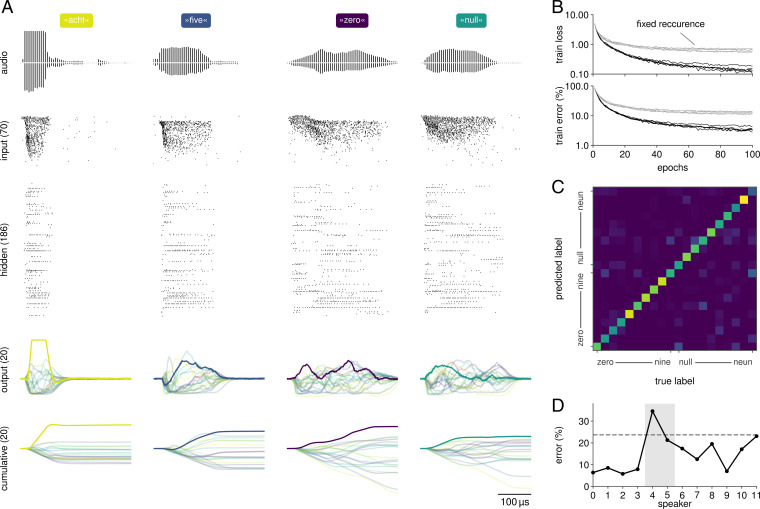
Classification of natural language with recurrent SNNs on BrainScaleS-2. (*A*) Responses of a recurrent network when presented with samples from the SHD dataset. The input spike trains, originally derived from recordings of spoken digits (illustrations), were reduced to 70 stimuli. The network was trained according to a sum-over-time loss based on the output units’ membrane traces. For visualization purposes, we also show their cumulative sums. (*B*) Over 100 epochs of training, the network developed suitable representations as evidenced by a reduced training loss and error, here shown for five distinct initial conditions. When training the network with fixed recurrent weights, it converges to a higher loss and error. (*C*) Classification performance varies across the 20 classes, especially since some of them exhibit phonemic similarities (»nine« vs. »neun«). (*D*) The trained network generalizes well on unseen data from most speakers included in the dataset. The discrepancy between training and overall test error (dashed line) arises from the composition of the dataset: 81% of the test set’s samples stem from two exclusive speakers (highlighted in gray).

To demonstrate successful learning of recurrent connections with our framework, we trained a network with 186 recurrently connected hidden-layer neurons on the Spiking Heidelberg Digits (SHD) dataset (36), which consists of spoken digits from »zero« to »nine« in both English and German, resulting in 20 classes total. This dataset is a natural benchmark for SNNs due to its inherent temporal dimension. Furthermore, it directly provides input spike trains and, hence, alleviates the need for additional preprocessing, which can confound comparison. To feed the data into our system, we reduced their dimensionality by subsampling 70 out of the original 700 channels (cf. *Materials and Methods*). The network was then trained by optimizing a sum-over-time loss $L=\text{NLL}(\text{softmax}({\text{sum}}_{t}\;V_{i}^{\text{O}}[t]),y^{⋆})$ (Fig. 5*A*). To prevent pathologically high firing rates, we employed homeostatic regularization during training. Specifically, we added a regularizer of the form $\rho_{\text{r}}\cdot \max{\left(0,\sum_{i,t}S_{i}[t]-\vartheta_{\text{r}}\right)}^{2}$, where *i* and *t* iterate over the hidden-layer units and time steps, respectively; $\rho_{\text{r}}$ defines the regularization strength; and $\vartheta_{\text{r}}$ an activity threshold.

After 100 training epochs, the SNN reached 96.6 ± 0.5% on the training data and 76.2 ± 1.3% on the test set (Fig. 5*B* and Table 1). The large gap is presumably due to the nature of the dataset, which was designed to especially challenge a network’s ability to generalize (36). The two languages included in the dataset exhibit classes with significant phonemic similarity (»nine« vs. »neun«), which are indeed harder to separate by the trained network (Fig. 5*C*). Most importantly, however, the test set consists to 81% of two speakers that are not part of the training set and result in higher classification error rates (Fig. 5*D*). To improve generalization performance, we employed data augmentation. For this purpose, we stochastically shifted events to neighboring input channels drawn from a normal distribution centered around their original channel (cf. *Materials and Methods*). This approach improved the test performance to 80.6 ± 1.0%.

To test whether good performance was dependent on learning of recurrent connections, we initialized a set of networks with the shuffled recurrent weights from a trained network and only trained its input and readout weights (Fig. 5*B*). This resulted in a substantial reduction of classification performance to 64.3 ± 2.3%. Thus, our ITL learning framework can leverage recurrent connections to improve accuracy on this benchmark.

To again compare our hardware system to simulations, we trained and evaluated the SNN in an equivalent software-only implementation. Without augmentation, it reached an accuracy of 71.2 ± 0.3%—far below the corresponding hardware results. At the same time, the software simulation was able to perfectly fit the training data, which was not achieved on BrainScaleS-2. We speculate that this discrepancy results from the intrinsic stochasticity of the analog substrate, which is propagated and amplified by the network’s recurrent dynamics and thereby acts as a form of regularization. When we applied the same data augmentation to the simulation as in our hardware emulation, this resulted in a improved test accuracy of 79.9 ± 0.7%, close to the performance of BrainScaleS-2 under equivalent conditions. Thus, our work suggests that intrinsic analog device noise could act as an efficient regularizer.

Finally, we compared the energy efficiency of our recurrent SNNs to recently published work on the Aloha keyword-spotting task. To that end, we trained a recurrent network with 176 hidden units on the task and found comparable performance at an energy efficiency competitive to Loihi and Movidius (ref. 37; *SI Appendix*, *SI Text* and Table S1).

In summary, our findings illustrate that the flexibility of ITL learning also applies to the realm of recurrent SNN trained on challenging speech-processing problems.

## Discussion

We have developed a general ITL learning method for recurrent and multilayer SNNs on analog neuromorphic substrates and demonstrated its capabilities on BrainScaleS-2. The combination of surrogate gradients with ITL training—facilitated by the massively parallel digitization of analog membrane potentials—allowed us to tie on recent achievements in the field of SNN optimization and bring them to an analog substrate. This allowed us to achieve state-of-the-art classification accuracies on multiple benchmark problems, comparable to equivalent software simulations. During training, our framework automatically corrected for device mismatch and thus abolished the need for explicit calibration. The resulting SNNs exhibited spatially and temporally sparse activity patterns and could furthermore be optimized for resilience to neuron failure. Ultimately, our method allowed us to exploit BrainScaleS-2 for low-latency neuromorphic inference at high throughput and a low energy footprint.

Most current neuromorphic systems are fully digital and typically allow one to simulate software-trained models without performance loss (38, 39). This approach is flexible with regard to the SNN training schemes used (23, 40–49), but to fully leverage recent advances in material sciences often requires dealing with analog or mixed-signal components (15, 19, 50, 51). For instance, memristors, a key emerging technology, are ideal candidates for long-term memory storage in neuromorphic systems (52–54). However, these respective components are intrinsically analog and subject to drift and manufacturing variability. These imperfections can reduce performance when loading software-trained models onto the analog substrate. While several studies approached this problem by optimizing for additional robustness during training (44, 55), these techniques are intrinsically limited. Since mature on-chip training solutions are not yet available, ITL learning has emerged as a good compromise that efficiently takes device-specific nonidealities and heterogeneity into account (56–59). However, previous work relied on rate-based or time-to-first-spike coding schemes. Here, we expanded ITL techniques into the realm of surrogate gradient learning, which flexibly interpolates between rate- and timing-based coding schemes on multilayer and recurrent architectures, thereby simultaneously improving performance and energy efficiency, while also being conducive for fast inference (24).

Comparing the performance of neuromorphic SNN implementations is an intricate task, starting with a lack of standardized benchmarks (36, 60). When surveying the broad spectrum of different neuromorphic architectures, one encounters diverse ways of determining a system’s energy consumption, which ranges from presilicon estimates of a neuromorphic core’s current draw to full-system laboratory measurements. Nevertheless, we attempted to contrast our findings with results from previous studies on both digital and analog systems (Table 2). Although lacking the essence of temporal spike-based information processing, we considered the MNIST dataset due to its widespread adoption.

**Table 2.** Comparison of MNIST benchmark results across neuromorphic platforms

	Platform	Reference	Architecture	Node, nm	Accuracy, %	Energy/inference, µJ	Throughput, inference×s^–1^	Latency, µs
Digital	SpiNNaker	Stromatias et al. (61)	784-500-500-10	130	95.0	/*	/*	/
	TrueNorth	Esser et al. (62)	CNN (1 ensemble)	28	92.7	0.27	1,000	/
			CNN (16 ensembles)	28	95	4	1,000	/
			CNN (64 ensembles)	28	99.4	108.0	1,000	/
	—	Chen et al. (63)	236-20	10	88.0	1.0	6,250	/
			784-1024-512-10	10	98.2	12.4	/	/
			784-1024-512-10	10	97.9	1.7	/	/
	MorphIC	Frenkel et al. (64)	784-500-10^†^	65	97.8	205	/	/
			784-500-10^†^	65	95.9	21.8	250	/
	SPOON	Frenkel et al. (38)	CNN	28	97.5	0.3^‡^	/	117
Analog	BSS-1	Schmitt et al. (56)	100-15-15-5	180	95.0	/*	10,000	/
	BSS-2	Göltz et al. (57)	256-246-10	65	96.9	8.4	21,000	¡10
	BSS-2	This work	256-246-10	65	97.6	2.4	85,000	8

* Estimates were given by Pfeiffer and Pfeil (40).

^†^Segmented input and hidden layers.

^‡^Based on presilicon estimates.

Our model on BrainScaleS-2 performed competitively in all metrics and was surpassed in accuracy only by much larger or convolutional networks. When considering the energy footprint, BrainScaleS-2 reached values only outperformed by optimized architectures fabricated in much smaller and hence more efficient technology nodes (38, 63). In comparison to other neuromorphic systems, we set benchmarks in terms of throughput and latency, which even challenge dedicated ANN accelerators (*SI Appendix*, Tables S1 and S2).

One limitation of our study is that, in addition to MNIST, we primarily used speech-based benchmark datasets to compare to other systems. Using accelerated systems such as BrainScaleS-2 for speech recognition would require an extra conversion step at the sensor level and thus likely result in additional energy costs that we did not quantify in our study. However, it is conceivable that part of this cost could be offset by dynamical network approaches and effective time-multiplexing strategies in edge applications or data centers. We primarily chose speech benchmarks due to the lack of suitable alternatives (60) and as a proxy for challenging problems requiring temporal processing that fits the number of channels supported by our system. While we expect that our main findings will generalize to other task domains and other neuromorphic substrates, showing this equivalence is left for future work.

In summary, our work shows how learning can efficiently compensate for device-specific imperfections, thereby allowing us to employ analog neuromorphic substrates for complex, energy-efficient, and ultralow-latency information processing. Importantly, it also is the first step toward future on-chip learning algorithms that could even take advantage of such device heterogeneity (65). Thus, our work gives a glimpse of how powerful learning algorithms will empower future neuromorphic technologies.

## Materials and Methods

### Software Environment

Our training framework was based on PyTorch’s autodifferentiation library (29). It furthermore builds upon the BrainScaleS-2 software stack to configure the neuromorphic system and execute the experiments (66).

### Input Coding

For MNIST, we scaled down the dataset to 16 × 16 pixels by first discarding the two outermost rows and scaling the remaining pixels. The images were then converted to spikes by interpreting the normalized pixel grayscale values *x_i_* as input currents to LIF neuons. Strong enough currents trigger a spike at time $t_{i}=\tau_{\text{in}}\log\;x_{i}/(x_{i}-\vartheta_{\text{in}})$, where $\tau_{\text{in}}$ denotes the input units time constant and $\vartheta_{\text{in}}$ its threshold (*SI Appendix*, Table S3).

Since the SHD dataset is provided in the form of input spike times, a custom conversion was not required. For SHD, we reduced the original 700 input channels by subsampling. Specifically, we omitted the first 70 and then retained every 9th input unit. The time dimension was scaled by a factor of 2,000 to account for the system’s acceleration factor of 1,000 and further shorten the experiment duration to reduce the computation burden on the host system. When employing data augmentation, a spike originally originating from input channel *i* was reassigned to a neighboring channel drawn from N(μ=i,σ). This augmentation was applied prior to downsampling the inputs.

### Initialization

We used Kaming’s initialization (67) for both the hidden- and output-layer weights. Specifically, weights were drawn from a normal distribution with zero mean and an SD of ${\hat{\sigma }}_{w}/\sqrt{N_{\text{H,L}}}$ (*SI Appendix*, Table S3).

### Weight Scaling

Weight values had to be scaled, rounded, and cropped to the neuromorphic system’s weight resolution of 7-bit signed integers resulting from merging two 6-bit synapse circuits. The exact scaling took into account analog bias currents and other technical parameters and was heuristically optimized to equalize the response of the analog neuronal circuits and the model dynamics of the computational graph.

Due to the absence of a threshold for the nonspiking output layer, its membrane traces could be scaled arbitrarily. For the MNIST classification, we adopted a dynamic weight scaling for the output weights by aligning the largest absolute weight value as represented in software to the maximum weight possible on the substrate.

### Energy Measurements

We separately measured the current draw of the full application-specific integrated circuit on the individual supply rails via INA219 current/power monitors from Texas Instruments, which were for that purpose placed on the system’s carrier board. The power readings were taken during the execution of the forward pass.

## Supplementary Material

Supplementary File

## Data Availability

There are no data underlying this work. Driver software and code examples are available in GitHub (https://github.com/fmi-basel/brainscales-2-surrogate-gradients).

## References

[1] V. Mnih ., Playing Atari with deep reinforcement learning. arXiv [Preprint] (2013). https://arxiv.org/abs/1312.5602 23 (Accessed 23 December 2021).

[2] D. Silver ., Mastering the game of go without human knowledge. Nature 550, 354–359 (2017). 29052630 10.1038/nature24270

[3] T. B. Brown ., Language models are few-shot learners. arXiv [Preprint] (2020). https://arxiv.org/abs/2005.14165 23 (Accessed 23 December 2021).

[4] P. Sterling, S. Laughlin, Principles of Neural Design (MIT Press, Cambridge, MA, 2015).

[5] C. Mead, Neuromorphic electronic systems. Proc. IEEE 78, 1629–1636 (1990).

[6] C. Mead, M. Ismail, Analog VLSI Implementation of Neural Systems (Springer Science & Business Media, 2012).

[7] G Indiveri ., Neuromorphic silicon neuron circuits. Front. Neurosci. 5, 73 (2011). 21747754 10.3389/fnins.2011.00073 PMC3130465

[8] C. S. T. Thakur ., Large-scale neuromorphic spiking array processors: A quest to mimic the brain. Front. Neurosci. 12, 891 (2018). 30559644 10.3389/fnins.2018.00891 PMC6287454

[9] C. D. Schuman ., A survey of neuromorphic computing and neural networks in hardware. arXiv [Preprint] (2017). https://arxiv.org/abs/1705.06963 23 (Accessed 23 December 2021).

[10] P. A. Merolla ., Artificial brains. A million spiking-neuron integrated circuit with a scalable communication network and interface. Science 345, 668–673 (2014). 25104385 10.1126/science.1254642

[11] B. V. Benjamin ., Neurogrid: A mixed-analog-digital multichip system for large-scale neural simulations. Proc. IEEE 102, 699–716 (2014).

[12] S. Furber, Large-scale neuromorphic computing systems. J. Neural Eng. 13, 051001 (2016). 27529195 10.1088/1741-2560/13/5/051001

[13] K. Boahen, A neuromorph’s prospectus. Comput. Sci. Eng. 19, 14–28 (2017).

[14] M. Davies ., Loihi: A neuromorphic manycore processor with on-chip learning. IEEE Micro 38, 82–99 (2018).

[15] K. Roy, A. Jaiswal, P. Panda, Towards spike-based machine intelligence with neuromorphic computing. Nature 575, 607–617 (2019). 31776490 10.1038/s41586-019-1677-2

[16] M. Mahowald, R. Douglas, A silicon neuron. Nature 354, 515–518 (1991). 1661852 10.1038/354515a0

[17] J. Schemmel ., “A wafer-scale neuromorphic hardware system for large-scale neural modeling” in Proceedings of the International Symposium on Circuits and Systems (ISCAS) (IEEE, Piscataway, NJ, 2010), pp. 1947–1950.

[18] E. Chicca, F. Stefanini, C. Bartolozzi, G. Indiveri, Neuromorphic electronic circuits for building autonomous cognitive systems. Proc. IEEE 102, 1367–1388 (2014).

[19] D. Marković, A. Mizrahi, D. Querlioz, J. Grollier, Physics for neuromorphic computing. Nat. Rev. Phys. 2, 499–510 (2020).

[20] S. Ambrogio ., Equivalent-accuracy accelerated neural-network training using analogue memory. Nature 558, 60–67 (2018). 29875487 10.1038/s41586-018-0180-5

[21] Y. Bengio, N. Léonard, A. Courville, Estimating or propagating gradients through stochastic neurons for conditional computation. arXiv [Preprint] (2013). https://arxiv.org/abs/1308.3432 (Accessed 23 December 2021).

[22] M. Courbariaux, I. Hubara, D. Soudry, R. El-Yaniv, Y. Bengio, Binarized neural networks: Training deep neural networks with weights and activations constrained to+ 1 or-1. arXiv [Preprint] (2016). https://arxiv.org/abs/1602.02830 (Accessed 23 December 2021).

[23] E. O. Neftci, H. Mostafa, F. Zenke, Surrogate gradient learning in spiking neural networks: Bringing the power of gradient-based optimization to spiking neural networks. IEEE Signal Process. Mag. 36, 51–63 (2019).

[24] S. Davidson, S. B. Furber, Comparison of artificial and spiking neural networks on digital hardware. Front. Neurosci. 15, 651141 (2021). 33889071 10.3389/fnins.2021.651141 PMC8055931

[25] B. Yin, F. Corradi, S. M. Bohté, Accurate and efficient time-domain classification with adaptive spiking recurrent neural networks. Nat. Mach. Intell. 3, 905–913 (2021).

[26] S. Billaudelle ., “Versatile emulation of spiking neural networks on an accelerated neuromorphic substrate” in Proceedings of the International Symposium on Circuits and Systems (ISCAS) (IEEE, Piscataway, NJ, 2020), pp. 1–5.

[27] A. Grübl, S. Billaudelle, B. Cramer, V. Karasenko, J. Schemmel, Verification and design methods for the brainscales neuromorphic hardware system. J. Signal Process. Syst 92, 1277–1292 (2020).

[28] J. Schemmel, S. Billaudelle, P. Dauer, J. Weis, Accelerated analog neuromorphic computing. arXiv [Preprint] (2020). https://arxiv.org/abs/2003.11996 (Accessed 23 December 2021).

[29] A. Paszke ., “Pytorch: An imperative style, high-performance deep learning library” in Advances in Neural Information Processing Systems, H. Wallach ., Eds. (Curran Associates, Inc., Red Hook, NY, 2019), **vol.** 32, pp. 8024–8035.

[30] S. Friedmann ., Demonstrating hybrid learning in a flexible neuromorphic hardware system. IEEE Trans. Biomed. Circuits Syst. 11, 128–142 (2017). 28113678 10.1109/TBCAS.2016.2579164

[31] F. Zenke, S. Ganguli, Superspike: Supervised learning in multilayer spiking neural networks. Neural Comput. 30, 1514–1541 (2018). 29652587 10.1162/neco_a_01086 PMC6118408

[32] Y. LeCun, L. Bottou, Y. Bengio, P. Haffner, Gradient-based learning applied to document recognition. Proc. IEEE 86, 2278–2324 (1998).

[33] D. P. Kingma, J. Ba, Adam: A method for stochastic optimization. arXiv [Preprint] (2014). https://arxiv.org/abs/1412.6980 (Accessed 23 December 2021).

[34] H. Xiao, K. Rasul, R. Vollgraf, Fashion-MNIST: A novel image dataset for benchmarking machine learning algorithms. arXiv [Preprint] (2017). https://arxiv.org/abs/1708.07747 (Accessed 23 December 2021).

[35] F. Zenke, T. P. Vogels, The remarkable robustness of surrogate gradient learning for instilling complex function in spiking neural networks. Neural Comput. 33, 899–925 (2021).3351332810.1162/neco_a_01367

[36] B. Cramer, Y. Stradmann, J. Schemmel, F. Zenke, The Heidelberg spiking data sets for the systematic evaluation of spiking neural networks. IEEE Trans. Neural Netw. Learn. Syst., 10.1109/TNNLS.2020.3044364 (2020). 33378266

[37] P. Blouw, X. Choo, E. Hunsberger, C. Eliasmith, “Benchmarking keyword spotting efficiency on neuromorphic hardware” in Proceedings of the 7th Annual Neuro-Inspired Computational Elements Workshop (Association for Computing Machinery, New York, 2019), pp. 1–8.

[38] C. Frenkel, J. D. Legat, D. Bol, “A 28-nm convolutional neuromorphic processor enabling online learning with spike-based retinas” in Proceedings of the International Symposium on Circuits and Systems (ISCAS) (IEEE, Piscataway, NJ, 2020), pp. 1–5.

[39] S. K. Esser ., Convolutional networks for fast, energy-efficient neuromorphic computing. Proc. Natl. Acad. Sci. U.S.A. 113, 11441–11446 (2016). 27651489 10.1073/pnas.1604850113 PMC5068316

[40] M. Pfeiffer, T. Pfeil, Deep learning with spiking neurons: Opportunities and challenges. Front. Neurosci. 12, 774 (2018). 30410432 10.3389/fnins.2018.00774 PMC6209684

[41] S. M. Bohte, J. N. Kok, H. La Poutre, Error-backpropagation in temporally encoded networks of spiking neurons. Neurocomputing 48, 17–37 (2002).

[42] B. Rückauer, N. Känzig, S. C. Liu, T. Delbruck, Y. Sandamirskaya, Closing the accuracy gap in an event-based visual recognition task. arXiv Preprint (2019). https://arxiv.org/abs/1906.08859 (Accessed 23 December 2021).

[43] D. Zambrano, R. Nusselder, H. S. Scholte, S. M. Bohté, Sparse computation in adaptive spiking neural networks. Front. Neurosci. 12, 987 (2019). 30670943 10.3389/fnins.2018.00987 PMC6332470

[44] J. Büchel, D. Zendrikov, S. Solinas, G. Indiveri, D. R. Muir, Supervised training of spiking neural networks for robust deployment on mixed-signal neuromorphic processors. Sci. Rep. 11, 23376 (2021). 34862429 10.1038/s41598-021-02779-x PMC8642544

[45] E. Hunsberger, C. Eliasmith, Spiking deep networks with LIF neurons. arXiv [Preprint] (2015). https://arxiv.org/abs/1510.08829 (Accessed 23 December 2021).

[46] J. H. Lee, T. Delbruck, M. Pfeiffer, Training deep spiking neural networks using backpropagation. Front. Neurosci. 10, 508 (2016). 27877107 10.3389/fnins.2016.00508 PMC5099523

[47] H. Mostafa, Supervised learning based on temporal coding in spiking neural networks. IEEE Trans. Neural Netw. Learn. Syst. 29, 3227–3235 (2018). 28783639 10.1109/TNNLS.2017.2726060

[48] G. Bellec ., A solution to the learning dilemma for recurrent networks of spiking neurons. Nat. Commun. 11, 3625 (2020). 32681001 10.1038/s41467-020-17236-y PMC7367848

[49] D. Huh, T. J. Sejnowski, “Gradient descent for spiking neural networks” in Advances in Neural Information Processing Systems, S. Bengio ., Eds. (Curran Associates, Inc., Red Hook, NY, 2018), vol. 31, pp. 1–11.

[50] V. Joshi ., Accurate deep neural network inference using computational phase-change memory. Nat. Commun. 11, 2473 (2020). 32424184 10.1038/s41467-020-16108-9 PMC7235046

[51] T. Dalgaty ., In situ learning using intrinsic memristor variability via Markov Chain Monte Carlo sampling. Nat. Electron. 4, 151–161 (2021).

[52] S. P. Adhikari, C. Yang, H. Kim, L. O. Chua, Memristor bridge synapse-based neural network and its learning. IEEE Trans. Neural Netw. Learn. Syst. 23, 1426–1435 (2012). 24807926 10.1109/TNNLS.2012.2204770

[53] Y. Kim, Y. Zhang, P. Li, “A digital neuromorphic VLSI architecture with memristor crossbar synaptic array for machine learning” in Proceedings of the International SOC Conference (IEEE, Piscataway, NJ, 2012), pp. 328–333.

[54] G. W. Burr ., Recent progress in phase-change memory technology. IEEE J. Emerg. Sel. Top. Circuits Syst. 6, 146–162 (2016).

[55] S. Moon, K. Shin, D. Jeon, Enhancing reliability of analog neural network processors. IEEE Trans. Very Large Scale Integr. VLSI Syst. 27, 1455–1459 (2019).

[56] S. Schmitt ., “Neuromorphic hardware in the loop: Training a deep spiking network on the brainscales wafer-scale system” in Proceedings of the International Joint Conference on Neural Networks (IJCNN) (IEEE, Piscataway, NJ, 2017), pp. 2227–2234.

[57] J. Göltz ., Fast and energy-efficient neuromorphic deep learning with first-spike times. Nat. Mach. Intell. 3, 823–835 (2021).

[58] P. Yao ., Fully hardware-implemented memristor convolutional neural network. Nature 577, 641–646 (2020). 31996818 10.1038/s41586-020-1942-4

[59] L. G. Wright ., Deep physical neural networks enabled by a backpropagation algorithm for arbitrary physical systems. arXiv [Preprint] (2021). https://arxiv.org/abs/2104.13386 (Accessed 23 December 2021).

[60] M. Davies, Benchmarks for progress in neuromorphic computing. Nat. Mach. Intell. 1, 386–388 (2019).

[61] E. Stromatias ., Robustness of spiking deep belief networks to noise and reduced bit precision of neuro-inspired hardware platforms. Front. Neurosci. 9, 222 (2015). 26217169 10.3389/fnins.2015.00222 PMC4496577

[62] S. K. Esser, R. Appuswamy, P. Merolla, J. V. Arthur, D. S. Modha, Backpropagation for energy-efficient neuromorphic computing. Adv. Neural Inf. Process. Syst. 28, 1117–1125 (2015).

[63] G. K. Chen, R. Kumar, H. E. Sumbul, P. C. Knag, R. K. Krishnamurthy, A 4096-neuron 1m-synapse 3.8-pJ/SOP spiking neural network with on-chip STDP learning and sparse weights in 10-nm FinFET CMOS. IEEE J. Solid-State Circuits 54, 992–1002 (2019).

[64] C. Frenkel, J. D. Legat, D. Bol, Morphic: A 65-nm 738k-synapse/mm^2^ quad-core binary-weight digital neuromorphic processor with stochastic spike-driven online learning. IEEE Trans. Biomed. Circuits Syst. 13, 999–1010 (2019). 31329562 10.1109/TBCAS.2019.2928793

[65] N. Perez-Nieves, V. C. H. Leung, P. L. Dragotti, D. F. M. Goodman, Neural heterogeneity promotes robust learning. Nat. Commun. 12, 5791 (2021). 34608134 10.1038/s41467-021-26022-3 PMC8490404

[66] E. Müller ., Extending brainscales OS for brainscales-2. arXiv [Preprint] (2020). https://arxiv.org/abs/2003.13750 (Accessed 23 December 2021).

[67] K. He, X. Zhang, S. Ren, J. Sun, “Delving deep into rectifiers: Surpassing human-level performance on ImageNet classification” in Proceedings of the International Conference on Computer Vision (IEEE, Piscataway, NJ, 2015), pp. 1026–1034.

