# Supplementary material for: Surrogate gradients for analog neuromorphic computing
Source: Proc Natl Acad Sci U S A. 2022 Jan 14;119(4):e2109194119. doi: 10.1073/pnas.2109194119 (PMC8794842; doi:10.1073/pnas.2109194119)
Supplement: Supplementary File [file pnas.2109194119.sapp.pdf]

Supplementary Information: Surrogate gradients for analog neuromorphic computing

Benjamin Cramer[a,1,2], **Sebastian Billaudelle**[a,1,2], **Simeon Kanya**[a], **Aron Leibfried**[a], **Andreas Grübl**[a], **Vitali Karasenko**[a], **Christian Pehle**[a], **Korbinian Schreiber**[a], **Yannik Stradmann**[a], **Johannes Weis**[a], **Johannes Schemmel**[a], **and Friedemann Zenke**[b]

[a]Kirchhoff-Institute for Physics, Heidelberg University, Germany; [b]Friedrich Miescher Institute for Biomedical Research, Basel, Switzerland

Aloha Keyword Spotting Benchmark

To compare our network models with other neuromorphic systems and ANN accelerators we used the Aloha keyword spotting benchmark dataset (1).

Audio preprocessing and conversion to currents. To convert raw audio to spikes, we preprocessed the raw audio signal $x(t)$ by first applying a pre-emphasis filter by computing $y(t) = x(t) - 0.95x(t-1)$. We then extracted 25ms frames with a 10ms stride from $y(t)$ to which we applied a Hamming window before computing 512-point fast Fourier transform. On the resulting power spectrum we further applied 40 triangular filters on a Mel-scale (2) and cropped and padded to a total of 80 time steps by repeating the last frame.

Current to spike conversion. To convert the resulting spectrograms into input spikes for our models, we interpreted the spectrogram channels as currents I_i which were fed into two adaptive leaky integrate-and-fire (LIF) neurons per input channel ($k = \{1, 2\}$). To that end, we simulated the following dynamics for synaptic currents G_i^k, membrane potentials U_i^k, and the adaptation variable A_i^k in discrete time:

$$G_i^k[t+1] = \alpha G_i^k[t] + w^k I_i\left[\lfloor \zeta t \rfloor\right] - b^k \quad [1]$$

$$U_i^k[t+1] = \beta U_i^k[t] + (1-\beta)\left(G_i^k[t] - aA_i^k[t]\right)\left(1.0 - S_i^k[t]\right) \quad [2]$$

$$A_i^k[t+1] = \gamma A_i^k[t] + S_i^k \quad [3]$$

where S_i^k is the output spike train, $w^k = \{0.02, 0.15\}$ is the input gain, $b^k = \{0.35, 0.2\}$ a bias term, $a = 0.1$ the adaptation strength, and $\zeta = 2.5$ a conversion factor that converts between the input current time grid to the LIF simulation time grid which was fixed at 200 time steps. An output spike S_i^k was generated whenever the corresponding voltage variable U_i^k crossed the firing threshold of 1. Moreover, we set the scaling variables $\alpha = \exp\left(-\Delta t/\tau_{\mathrm{syn}}\right)$, $\beta = \exp\left(-\Delta t/\tau_{\mathrm{mem}}\right)$, and $\gamma = \exp\left(-\Delta t/\tau_{\mathrm{ada}}\right)$ with $\Delta t = 2$ms,

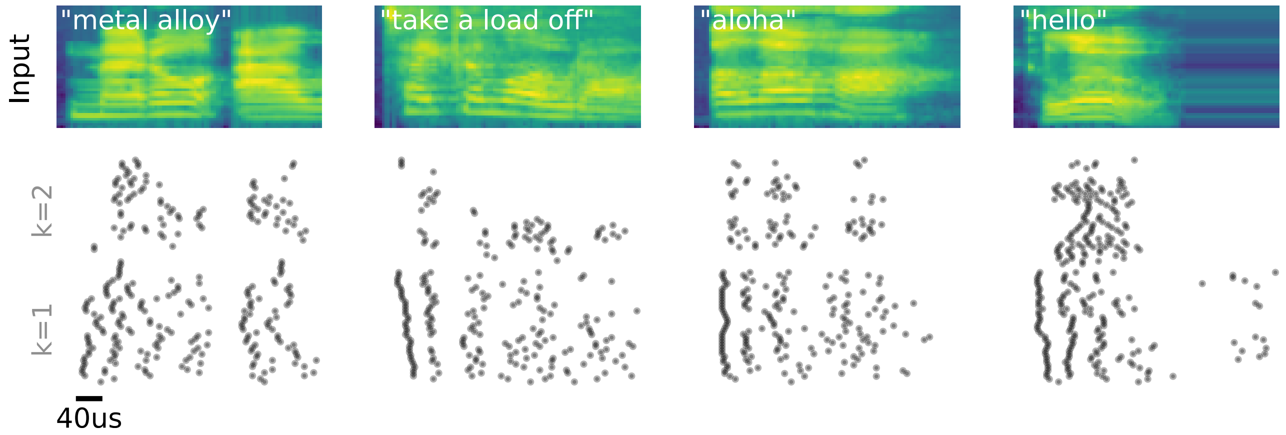

Supplementary Figure 1. Four example inputs from the Aloha keyword spotting benchmark. Input currents (top) and spike raster plots (bottom). Note that we plotted the encoder neurons sorted by $k = \{1, 2\}$.

$\tau_{\mathrm{syn}} = 5\mathrm{ms}$, $\tau_{\mathrm{mem}} = 10\mathrm{ms}$, and $\tau_{\mathrm{ada}} = 100\mathrm{ms}$. Finally, the resulting sparse spiking activity was compressed in time by the $1000\times$ hardware acceleration factor (Supplementary Fig. 1) prior to feeding them into the BrainScaleS-2 system.

Training and regularization. We trained our recurrent spiking neural networks (SNNs) in analogy to the ones optimized for the spiking Heidelberg digits (SHD) dataset. To improve the network's ability to generalize on unseen data, input spikes were dropped with a probability of 8 % during training. While Blouw et al. (1) paired letter recognition in the spiking network with a subsequent in-software pattern matching on the following allowed strings `['loha', 'alha', 'aloa', 'aloh', 'aoha', 'aloha']`, we trained our system end-to-end on recognizing the entire "aloha" keyword directly.

Keyword spotting results. On the Aloha keyword spotting benchmark, our trained networks reached a true positive rate of (89.6 ± 3.3) % and a true negative rate of (97.9 ± 0.8) %, measured across ten networks trained on different sets of initial conditions (Supplementary Table 1). Our best network classified the test data with a true positive rate of 92.7 % and a true negative rate of 99.0 %. These results are comparable with the error rates reported by Blouw et al. (1). The raw energy efficiency and throughput figures are, unfortunately, not directly comparable due to the dramatic differences in architecture and benchmarking methodology. For once, Blouw et al. (1) based their energy figures only on the systems' dynamic power; in our case, we also included the idle consumption, as it dominated the total numbers. For the throughput measurements, the authors included the overheads introduced by the Python-based host software and the communication to the respective devices. We instead measured the throughput of our ASIC instead, since in our case, the final classification was obtained on the embedded processor instead of the host system. The largest discrepancy, however, stems from the vastly different network architectures. While Blouw et al. (1) used a feedforward architecture with a CTC loss, we rely on a SNN with a standard Softmax cross entropy loss (3). In their measurement, (1), considered a single forward pass of their network, which corresponds to the classification of a single 10 ms time frame, whereby each keyword/phrase consists of 65 time windows on average. These measurements thus have to be understood as energy and throughput per frame. In contrast, we measured inference on complete keywords/phrases using our recurrent architecture and, consequently, report energy and throughput per inference.

References

1. Blouw P., Choo X., Hunsberger E., and Eliasmith C.. Benchmarking keyword spotting efficiency on neuromorphic hardware. In *Proceedings of the 7th Annual Neuro-inspired Computational Elements Workshop*, pages 1–8, 2019.
2. Huang X., Acero A., Hon H.-W., and Reddy R.. *Spoken Language Processing: A Guide to Theory, Algorithm and System Development.* Prentice Hall, 2001. ISBN 978-0-13-022616-7.
3. Cramer B., Stradmann Y., Schemmel J., and Zenke F.. The heidelberg spiking data sets for the systematic evaluation of spiking neural networks. *IEEE Transactions on Neural Networks and Learning Systems*, 2020.
4. Stromatias E., Neil D., Pfeiffer M., Galluppi F., Furber S. B., and Liu S.-C.. Robustness of spiking deep belief networks to noise and reduced bit precision of neuro-inspired hardware platforms. *Frontiers in Neuroscience*, 9:222, 2015. .

Supplementary Table 1. Comparison of the Aloha Keyword Spotting Benchmark results across neuromorphic and conventional platforms.

platform	architecture	true positive (%)	true negative (%)	energy/frame (μJ)	throughput (Frame s^{-1})	energy/inference (μJ)	throughput (Inference s^{-1})
CPU(1)	390-256-256-29 [i]	92.7	97.9	6300 [iii]	1813		
GPU(1)		92.7	97.9	29 800 [iii]	770		
Jetson(1)		92.7	97.9	5600 [iii]	419		
Movidius(1)		92.7	97.9	1500 [iii]	300		
Loihi(1)		93.8	97.9	270 [iii]	296		
BSS-2 (this work)	80-176-2 (recurrent)	89.6 ± 3.3 [ii]	97.9 ± 0.8 [ii]			70 [iv]	2800

[i] Inference involves additional post-processing on the host system to obtain the final classification result. [ii] We observed strong fluctuations in performance for different initial conditions. Our best network evaluated to a true positive rate of 92.7 % and a true negative rate of 99.0 %. [iii] Calculated from dynamic power consumption. [iv] Calculated from total power consumption.

Supplementary Table 2. Comparison of MNIST results across spike-based neuromorphic platforms and ANN accelerators.

	platform	reference	architecture	node (nm)	accuracy (%)	energy/inference (μJ)	throughput (Inference s^{-1})	latency (μs)
digital	SpiNNaker	Stromatias et al.(4)	784-500-500-10	130	95.0	/ [iii]	/ [iii]	/
	TrueNorth	Esser et al. (5)	CNN (1 ensemble)	28	92.7	0.27	1000	/
			CNN (16 ensembles)	28	95	4	1000	/
			CNN (64 ensembles)	28	99.4	108.0	1000	/
	—	Chen et al. (6)	236-20	10	88.0	1.0	6250	/
			784-1024-512-10	10	98.2	12.4	/	/
			784-1024-512-10	10	97.9	1.7	/	/
	MorphIC	Frenkel et al. (7)	784-500-10[i]	65	97.8	205	/	/
			784-500-10[i]	65	95.9	21.8	250	/
	SPOON	Frenkel et al. (8)	CNN	28	97.5	0.3 [ii]	/	117
analog	BSS-1	Schmitt et al. (9)	100-15-15-5	180	95.0	/ [iii]	10 000	/
	BSS-2	Göltz et al. (10)	256-246-10	65	96.9	8.4	21 000	< 10
	BSS-2	this work	256-246-10	65	97.6	2.4	85 000	8
ANN	BinarEye	Moons et al. (11)	CNN (9 layers)	28	98.85	14.4	120	8333 [iv]
			CNN (9 layers)	28	97.50	3.47	500	2000 [iv]
			CNN (9 layers)	28	96.70	0.92	1700	588 [iv]
			CNN (5 layers)	28	97.4	0.21	/	/
	DNN Engine	Whatmough et al. (12)	784-256-256-256-10	28	98.4	0.57	111 000	/
			284-256-256-256-10	28	98.4	0.36	61 000	/
	—	Chen et al. (13)	128-1024-10	65	93.7	0.11	14 300	/

5. Esser S. K., Appuswamy R., Merolla P., Arthur J. V., and Modha D. S.. Backpropagation for energy-efficient neuromorphic computing. *Advances in neural information processing systems*, 28: 1117–1125, 2015.
6. Chen G. K., Kumar R., Sumbul H. E., Knag P. C., and Krishnamurthy R. K.. A 4096-neuron 1m-synapse 3.8-pj/sop spiking neural network with on-chip stdp learning and sparse weights in 10-nm finfet cmos. *IEEE Journal of Solid-State Circuits*, 54(4):992–1002, 2019.
7. Frenkel C., Legat J.-D., and Bol D.. Morphic: A 65-nm 738k-synapse/mm² quad-core binary-weight digital neuromorphic processor with stochastic spike-driven online learning. *IEEE transactions on biomedical circuits and systems*, 13(5):999–1010, 2019.
8. Frenkel C., Legat J.-D., and Bol D.. A 28-nm convolutional neuromorphic processor enabling online learning with spike-based retinas. In *2020 IEEE International Symposium on Circuits and Systems (ISCAS)*, pages 1–5. IEEE, 2020.
9. Schmitt S., Klähn J., Bellec G., Grübl A., Guettler M., Hartel A., Hartmann S., Husmann D., Husmann K., Jeltsch S., et al. Neuromorphic hardware in the loop: Training a deep spiking network on the brainscales wafer-scale system. In *2017 International Joint Conference on Neural Networks (IJCNN)*, pages 2227–2234. IEEE, 2017.
10. Göltz J., Baumbach A., Billaudelle S., Breitwieser O., Dold D., Kriener L., Kungl A. F., Senn W., Schemmel J., Meier K., et al. Fast and deep neuromorphic learning with time-to-first-spike coding. *arXiv preprint arXiv:1912.11443*, 2019.
11. Moons B., Bankman D., Yang L., Murmann B., and Verhelst M.. Binareye: An always-on energy-accuracy-scalable binary cnn processor with all memory on chip in 28nm cmos. In *2018 IEEE Custom Integrated Circuits Conference (CICC)*, pages 1–4. IEEE, 2018.
12. Whatmough P. N., Lee S. K., Brooks D., and Wei G.-Y.. Dnn engine: A 28-nm timing-error tolerant sparse deep neural network processor for iot applications. *IEEE Journal of Solid-State Circuits*, 53(9):2722–2731, 2018.
13. Chen Y., Wang Z., Patil A., and Basu A.. A 2.86-tops/w current mirror cross-bar-based machine-learning and physical unclonable function engine for internet-of-things applications. *IEEE Transactions on Circuits and Systems I: Regular Papers*, 66(6):2240–2252, 2019.

Supplementary Table 3. Parameters for the neuromorphic substrate and learning framework.

parameter	value (MNIST / SHD)
difference threshold-leak $\vartheta - V_{\mathrm{leak}}$	(270 ± 15) mV
membrane time constant τ_{m}	(5.7 ± 0.3) µs / (8.6 ± 1.2) µs
in computation graph	6.0 µs / 10.0 µs
synaptic time constant τ_{s}	(6.5 ± 0.1) µs / (11.2 ± 0.5) µs
in computation graph	6.0 µs / 10.0 µs
input unit time constant τ_{in}	8 µs / –
input unit threshold ϑ_{in}	0.2 / –
surrogate gradient steepness β	50
learning rate η	1.5×10^{-3}
learning rate decay per epoch γ_η	0.03 / 0.025
amplitude regularization strength ρ_{a}	4×10^{-4} / –
burst regularization strength ρ_{b}	0.005 / –
rate regularization strength ρ_{r}	– / 0.6×10^{-3}
rate regularization threshold ϑ_{r}	– / 600
time step/sample period Δt	1.7 µs
weight initialization spread $\hat{\sigma}_w$	0.17, 0.34 for recurrent
